# Determinants of Differences in Health Service Utilization between Older Rural-to-Urban Migrant Workers and Older Rural Residents: Evidence from a Decomposition Approach

**DOI:** 10.3390/ijerph19106245

**Published:** 2022-05-20

**Authors:** Li Li, Jinjuan Yang, Shaoguo Zhai, Dan Li

**Affiliations:** 1 School of Public Management, Northwest University, Xi’an 710127, China; lililuckyball@nwu.edu.cn (L.L.); dandanli@nwu.edu.cn (D.L.); 2 School of Public Health, Health Science Center, Xi’an Jiaotong University, Xi’an 710061, China; yangjinjuan@stu.xjtu.edu.cn

**Keywords:** determinants of differences, health service utilization, older rural-to-urban migrant workers, Fairlie’s decomposition

## Abstract

Background: The widening gap in health service utilization between different groups in mainland China has become an important issue that cannot be avoided. Our study explored the existence of differences and the causes of the differences in the health service utilization of older rural-to-urban migrant workers in comparison to older rural dwellers. Further, our study explored socioeconomic differences in health service utilization. Methods: The data from the China Labor-Force Dynamic Survey in 2016, the data from the Urban Statistical Yearbook in 2016, and the Statistical Bulletin were used. Our study applied the latest Andersen Model according to China’s current situation. Before we studied health service utilization, we used Coarsened Exact Matching to control the confounding factors. After matching, 2314 respondents were successfully matched (859 older rural-to-urban migrant workers and 1455 older rural dwellers). The Fairlie decomposition method was used to analyze the differences and the sources of health service utilization between older rural-to-urban migrant workers and their rural counterparts. Results: After matching, the probability two-weeks outpatient for older rural-to-urban migrant workers (5.59%) was significantly lower than older rural dwellers (7.57%). The probability of inpatient for older rural-to-urban migrant workers (5.59%) was significantly lower than older rural dwellers (9.07%). Overall, 17.98% of the total difference for two-week outpatient utilization was due to the observed influence factors. Moreover, 71.88% of total difference in inpatient utilization was due to the observed influence factors. Income quantiles (49.57%), health self-assessments (80.91%), and the sex ratio in the community (−102.29%) were significant in the differences in inpatient utilization. Conclusions: The findings provide important insights into the socioeconomic differences in health service utilization among older rural-to-urban migrant workers and older rural residents in China. These insights urge the government to take full account of the heterogeneity in designing health security system reform and public health interventions targeting vulnerable groups.

## 1. Background

Rural-to-urban migrant workers have made great contributions to the development of urbanization and industrialization in China. Aging and migration have significantly shaped the composition of the population in China, where older rural-to-urban migrant workers (age 50 and above) make up increasingly large proportions of the population [1,2,3]. According to the National Bureau of Statistics, the proportion of older rural-to-urban migrant workers in total rural-to-urban migrant workers had increased from 17.9% to 35.9% during the period of time from 2015 to 2019 [1]. Social Insurance Law in China stipulates that employers should pay the premiums for the social welfare insurance of full-time and part-time rural-to-urban migrant workers, including old-age, unemployment, and medical insurance; whereas plenty of older rural-to-urban migrant workers have not benefited from the statutory social security system because of the irresponsibility of employers.

Older rural-to-urban migrant workers are referred to as “the browning of the greying” in many countries [2]. It is not understood how the apparently greater differences in migration for work may affect the health service utilization of older rural-to-urban migrant workers. Increasingly, studies [3,4,5] have mentioned the healthy migrant effect, which is a healthy selection effect due to the substantial physical and mental demands of migration. The studies have drawn attention to older rural-to-urban migrant workers, and a common finding showed that, compared to older rural residents, older rural-to-urban migrant workers overall seem to suffer from chronic diseases and mental orders. Conversely, there is a shortage of research on the discussion of their health service utilization, not to mention the comparison of the health service utilization between older rural-to-urban migrant workers and older rural residents. To the best of our knowledge, only Zhao et al. explored the determinants of the four-week outpatient rate and the outpatient costs of rural-to-urban migrant workers aged 45 years and older [6]. Based on the assumption of Stark et al. [7], migration for work meant an investment that entails costs as well as benefits, including better health care. What needs further investigation is the explanatory mechanism for the health service utilization of older rural-to-urban migrant workers. 

Access to health services for legal immigrants is highly ranked on the policy agenda in most countries of the world. For example, compared to native Spanish residents, non-Spaniards seemed to face substantial entry barriers to specialized health care [8]. African immigrants were 73% less likely to have a regular health care provider than African American women because African immigrants face more unique barriers to access health care [9]. Several studies [10] have explored the difference in health service utilization between the residents and migrant population and its impact factors, and the evidence made it quite clear that compared to a migrant population, the rate of awareness regarding health recording and the completion rate of the health records of native residents were significantly higher. The migrant population in China, whose original intention for relocation was for the care of grandchildren, housekeeping, better health services, and family care, are different from Chinese migrant labors, older rural-to-urban migrant workers. A host of studies [3,4,11] suggested that migrant workers were likely to be a “selected” population with good health prior to migration. In their own way, older rural-to-urban migrant workers would generally rely on health services less than the original residences in the local population. In addition, during the period of aging, it is important to neutralize the effects of progressive involution changes in order to ensure comfort and quality of life, as well as to maintain optimal functional fitness for the next stages of old age [12]. Migration to cities for work represented a change in status for both older rural-to-urban migrant workers and older rural residents; that is, older rural-to-urban migrant workers move out, while older rural residents move in. Moreover, migration for work in cities is not randomly assigned because there generally is “self-selection based upon personal circumstances” [13]. Although prior studies have drawn more attention to the differences in the health service utilization of migrant populations relative to the residents from the perspective of the dual structure of urban and rural areas, very few studies have drawn attention to the comparison of health service utilization for older rural-to-urban migrant workers in China relative to their rural counterparts, not to mention the probable determinants of socioeconomic differences in their health service utilization.

The Anderson model provided a useful theoretical analysis framework for explaining an individual’s health service utilization [14]. Various studies have been conducted on the probable determinants at multiple levels of the original version of the Andersen model and later versions using three dimensions, including predisposing, enabling, and need variances [14,15]. The lasted Andersen model emphasized the dynamics and circularity of each dimension index from the perspective of systemics [16]. Nevertheless, there are few empirical studies that have applied the predictors of the latest Andersen model to grasp the latest development of the Andersen model in China [17,18].

To consider these patterns, this paper aimed to show a broader picture in terms of the health service utilization and the determinants of older rural-to-urban migrant workers and rural residents in China by using a nationally representative dataset and further exploring the causes of the socioeconomic difference in multiple levels of the latest Andersen model. Our hypothesis is that older rural migrant workers have a lower use of health services than the rural dwellers of the same age, and the influencing factors of their health service utilization and the determinants of differences among the two groups were diversified.

## 2. Methods

### 2.1. Data

**Figure 1:**
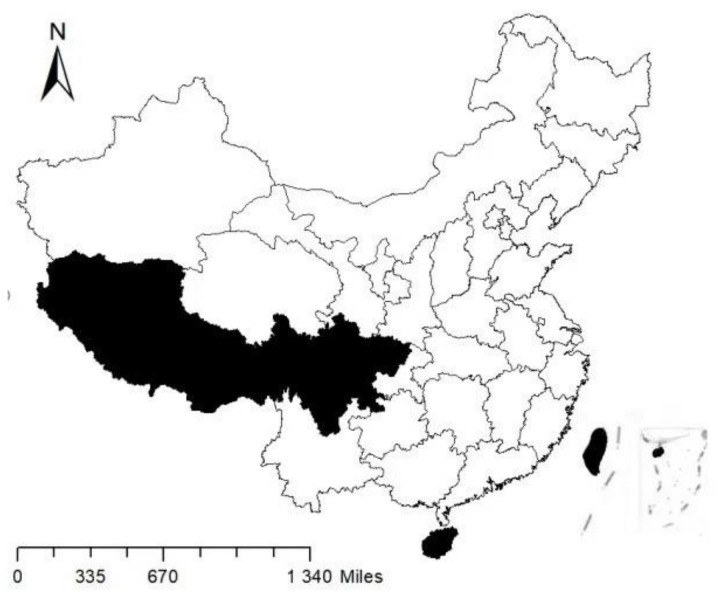
Map of provinces, municipalities and autonomous regions of China in CLDS 2016 survey. Note: The blank indicated the provinces, municipalities and autonomous regions of China surveyed in CLDS 2016, and the black indicates the provinces, municipalities and autonomous regions of China not surveyed in CLDS 2016.

The data included two parts. First, the socio-economic data for our study were obtained from the China Labor-Force Dynamic Survey in 2016 (CLDS 2016), which was issued by the Center for Social Survey at the Sun Yat-sen University. The CLDS 2016 was conducted across 29 provinces, municipalities, and autonomous regions in China (except Hong Kong, Macao, Taiwan, Tibet, and Hainan). The map of the Chinese provinces, municipalities, and autonomous regions surveyed in the CLDS 2016 survey is shown in Figure 1. Overall, 21,086 participants aged 15–64 were interviewed. This data set includes detailed accounts of demographic, health, economic, and health service utilization data. Second, we used data released by the Chinese government, which are reliable for obtaining indicators of cities and communities. Resource allocation in Chinese cities always has the characteristics of time delay and time accumulation [19,20]. Therefore, the data on the cities were obtained from the Urban Statistical Yearbook and the Statistical Bulletin of municipal governments published in 2015, and these data were released by the Chinese government and are reliable in regards to 2016.

### 2.2. Inclusion and Exclusion Criteria

We grouped cases on the basis of migration status and employment status, distinguishing older rural-to-urban migrant workers and older rural residents. In accordance with the Provisions on Statistical Division of Urban and Rural areas (Trial) [21] issued by China’s National Bureau of Statistics, cities and towns refer to the areas that have been established with the approval of the State Council in China, including the areas divided into districts and areas that are not divided into districts. Village refers to areas other than cities and towns. The urban and rural areas discussed in this study are inseparable from the dissimilation of the function of the Chinese household registration system (Hukou). The inclusion criteria were: aged 50~65; with rural Hukou (Chinese household registration system); a permanent residency in cities or towns; and employment of more than 6 months. At the same time, older rural residents, who are the same age as older rural-to-urban migrant workers, are also facing the situation of aging. Older rural residents who met the criteria were included in our study. The inclusion criteria were: aged 50~65; with rural Hukou; permanent residency in a village; unemployed or employed for less than 6 months. In our study, we restricted the age to 50 to 65 [4,5] to exclude those who have exited the labor market.

### 2.3. Measurements

According to the relevant literature [22,23,24], there are two kinds of indicators of health services utilization: (1) based on the actual health needs, including the utilization of the two-week outpatient system, inpatient utilization, hospitalization days, etc.; (2) the efficiency of the use of health resources, including the average annual number of patients received by each outpatient doctor, the utilization rate of hospital beds, etc. Our study measured the health service utilization by focusing on two-week outpatient and inpatient utilization. According to the accessible data in the CLDS 2016 questionnaire, the health service utilization can be represented by two-week visitation to a clinic (a person visited the clinic at least one time within two weeks) and by admissions to a hospital during the past 12 months when the respondent was sick or injured. They were dichotomized into binary variables (0-non-use and 1-use).

### 2.4. Predictor

**Figure 2:**
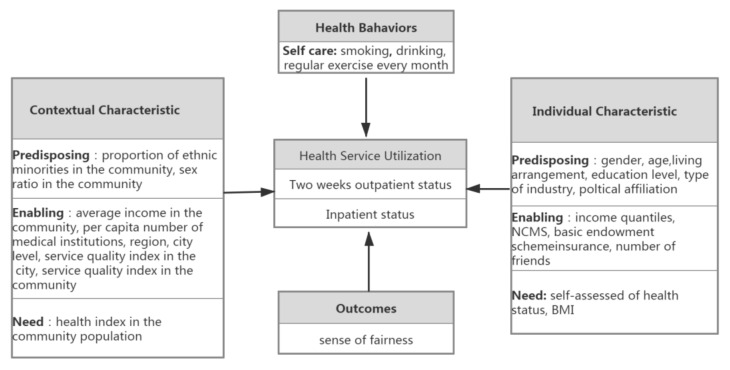
Revised Andersen Model (with appropriate citation).

Our study did not focus on the multiple interactions of four dimensions, and we only focused on the one-way relationship, that is, how the contextual characteristics, individual characteristics, health behaviors, and health outcomes affect health service utilization. Therefore, we simplified the analysis framework, and our study was primarily concerned with the one-way relationship; that is, health service utilization is determined by four dynamics: contextual characteristics, individual characteristics, health behaviors, and health outcomes. In addition, we selected predictors based on the latest Andersen model and the Model for Vulnerable Populations [25]. Combined with the purposes of this study and the availability of data, the determinants of health service utilization are shown in Figure 2.

Combined with the purposes of our study and the availability of data, four dimensions in our study can be constructed from the following aspects:

First, individual characteristics: age group (50~60; 61 and above), gender (male; female), living arrangement (living with spouse or living without a spouse), educational level (below primary school, primary school, middle school, and above), political affiliation (party member or unaffiliated), type of industry (manufacturing and construction, wholesale, retail trade and catering, transportation, and other non-agricultural sectors), place of work (local or out-of-town), working hours (moderate labor or excessive labor), New Cooperative Medical Scheme (NCMS) (yes or no), basic endowment scheme (yes or no), income quantiles (poorest, poorer, middle, richer, and richest), self-assessed health status (SAH) (good, fair, or poor), number of friends (≤5, 6~10, or ≥11).

Second, health behaviors: smoking (yes or no), drinking (yes or no), and regular exercise every month (yes or no).

Third, health outcomes: sense of fairness (unhappy, fair, or happy).

Fourth, contextual characteristics: the proportion of ethnic minorities, number of health facilities per capita in the community, sex ratio in the community, the service quality index of the community, the service quality index of the city, health index of the community population, region (east, middle, or west), and city level (sub-provincial city and above or below sub-provincial city). Among them, the service quality index of the community, the service quality index of the city, and the health index of the community population were constructed by factor analysis.

### 2.5. Coarsened Exact Matching (CEM)

A crude comparison of the health service utilization simply using multivariate models for each group would be more likely to neglect the difference in the population composition between the two groups and break the assumption that there is no bias present in the data [26,27]. Our study tackled a methodological issue in assessing the “causal effect” on the change in status by CEM because older rural-to-urban migrant workers and rural counterparts can become (or very close to) identical in relation to individual characteristics after CEM [3,26]. CEM controls for the effects, allowing our analysis to draw more attention to the change in status (migration for work), which would provide scientific evidence on the main difference in health service utilization between older rural-to-urban migrant workers and older rural residents. In our study, the employment status (be employed outside of the country for 6 months or more in the past year) was matched.

Compared to other matching methods, CEM can provide lower variance and bias for any sample size to improve causal inferences [28,29,30,31]. Iacus pointed out that more variables in the matching process would interfere with the exact matches, and he also proposed that robustness checks and robustness tests after CEM are not necessary [28]. Therefore, our study did not include all of the variables in our conceptual framework, and our study did not implement robustness checks after CEM. Age, gender, educational level, economic level, SAH, and BMI were used for matching in the process of CEM, and the matching weights generated by CEM were used to equalize the number within the comparison groups [28,29]. For the balance checking of the two comparison groups, the multivariate imbalance measure L1 was employed to measure the quality of the matching process, of which size depends on the data set and the selected covariates. The L1 ranges from 0–1, with 0 indicating a perfect global balance between the comparison groups and 1 indicating a maximal imbalance. A larger value represents a larger imbalance, and a substantial reduction in L1 indicated good matching performance [28,29]. CEM is an ado command by Blackwell, not an official Stata command, and CEM can be implemented with the “cem” command code in Stata15.0 (StataCorp LP., College Station, TX, USA) [32].

### 2.6. Fairlie Decomposition

Since the outcomes of interest in our study were binary, we utilized the decomposition technique proposed by Fairlie, which can solve the issues of the models, such as the logit and probit models [4,33,34]. The Fairlie decomposition method allows the identification of the observed differences between the two groups with differing employment status, and if differences are observed, the extent to which the contribution to the difference in health service utilization is. Fairlie decomposition method for a nonlinear equation, $Y=F(X\hat{\beta })$, can be written as follows [33,34]:

(1) $${\bar{Y}}^{w}-{\bar{Y}}^{B}=\left[\sum_{i=1}^{N^{w}}\frac{F(X_{i}^{w}{\hat{\beta }}^{w})}{N^{w}}-\sum_{i=1}^{N^{B}}\frac{F(X_{i}^{B}{\hat{\beta }}^{w})}{N^{B}}\right]+\left[\sum_{i=1}^{N^{B}}\frac{F(X_{i}^{B}{\hat{\beta }}^{w})}{N^{B}}-\sum_{i=1}^{N^{B}}\frac{F(X_{i}^{B}{\hat{\beta }}^{B})}{N^{B}}\right]$$

where $N^{j}$ is the sample size for group *j*. In (1), the first bracket represents the differences in the distributions of *X*, and the second represents the differences in the group processes, determining levels of Y. The first bracket represents the outcome differential due to the observable variables between the cohorts. The second bracket represents differences that can be interpreted as unobserved heterogeneity between the cohorts. The positive contribution of one factor indicated that the factor widened the differences and vice versa.

## 3. Result

### 3.1. Matching Performance

The characteristics of the respondents with and without weights are described in Table 1. It was obvious that there were significant differences in many characteristics between older rural-to-urban migrant workers and older rural dwellers before CEM, indicating that differences came from the identity of these groups. Overall, 2314 respondents were successfully matched by CEM (859 older rural-to-urban migrant workers and 1455 older rural dwellers). A statistical decrease in the *p*-value indicated a good matching performance. The L1 from 0.6372 to close to zero also revealed good matching performance. After CEM, the probability of two-weeks outpatient for older rural-to-urban migrant workers (5.59%) was significantly lower than that of older rural dwellers (8.11%). The probability of inpatient of older rural migrant workers (7.57%) was significantly lower than older rural dwellers (9.07%).

### 3.2. Logit Regression Analysis

As Table 2 shows, the statistically significant factors influencing the two-weeks outpatient utilization of older rural-to-urban migrant workers were age, self-assessed health, and city level of residency. The statistically significant factors influencing the inpatient service utilization of older rural dwellers were income quantiles, self-assessed health, BMI, and a sense of fairness. The statistically significant factors influencing the inpatient service utilization of older rural-to-urban migrant workers were the place of work, self-assessed health, regular exercise every month, and a sense of fairness. The statistically significant factors influencing the inpatient service utilization of older rural dwellers were educational attainment, self-assessed health, number of friends, and the service quality index of the community.

### 3.3. Fairlie’s Decomposition of Differences in Health Service Utilization

Table 3 further highlights that the two-week outpatient utilization and inpatient utilization of older rural-to-urban migrant workers were lower than older rural dwellers (total gaps were −0.0082 and −0.0106, respectively). The 17.98% of the total difference for the two-week outpatient utilization between the older rural-to-urban migrant workers and older rural dwellers was enlightened by the observed influence factors. Furthermore, 71.88% of the total difference for the inpatient utilization between older rural-to-urban migrant workers and older rural dwellers was due to the observed influence factors.

## 4. Discussion

Our study provided new empirical evidence on older rural-to-urban migrant workers (age 50 and above) in China, a group at the bottom of the heap in China. There is a tendency to treat the experience of older rural-to-urban migrant workers as homogeneous, to speak in broad generalities, ignoring differences in choice sets and potential outcomes of choices generated by geographical, cultural, and other differences. To the best of our knowledge, our study was the first large-scale comparative study on the health service utilization between older rural-to-urban migrants and older rural dwellers in China. In addition, a minor revision of the latest Andersen model in the socio-cultural context of China can provide theoretical support for the systematic explanation of the health service utilization of older rural-to-urban migrant workers and their rural counterparts in China.

What is actually happening behind their health service is that a small proportion of older rural-to-urban migrant workers were more likely to return to their place of origin for cheaper treatment when they suffered from diseases, and the groups were called returning migrant workers. Contradicting previous studies [7], we believed that rural-to-urban migrants were not clear about the policies on the off-site medical treatment and had insufficient knowledge of their health status. Indeed, the Migrant Population Service Center in China exerts efforts to provide health management for rural-to-urban migrant workers. Nevertheless, as a matter of fact, the cultural values and norms concerning their health care service use would constitute a barrier to raising their awareness of available health services.

Our results showed that the probability of two-week outpatient utilization and the probability of inpatient utilization by older rural-to-urban migrant workers was 5.59% and 7.57%, respectively, which were lower than the 8.11% and 9.07% of older rural dwellers. These findings are consistent with our hypothesis. The Analysis Report of the National Health Service Survey in China [24] showed that the probability of two-week outpatient utilization by older rural-to-urban migrant workers was far less than urban and rural residents (5.59% and 8.10%, respectively) and that the probability of inpatient older rural-to-urban migrant workers was far less than urban and rural residents (7.75% and 7.80%, respectively). By comparison with older rural dwellers, the results showed that older rural-to-urban migrant workers were at a disadvantage in health service utilization. Several following reasons could partially explain this. On the one hand, limited medical insurance and the reimbursement policy of off-site medical treatment made their health service utilization a luxury rather than a necessity. Low-level income and poor insurance coverage would cause most of the rural-to-urban migrant workers to be in poorer health because they could only afford a very limited level of the higher quality health care system that most of the older rural dwellers are turning to. In addition, older rural-to-urban migrant workers are often laid off in the event of an accident, and medical costs are not covered. As such, an enormous number of older rural-to-urban migrant workers may seek out self-healing or cease medical treatment or hospitalization. On the other hand, being employed was associated with less likelihood of health service utilization for older rural-to-urban migrant workers. Ordinarily, older rural migrant workers are a group of “healthy selection”, in line with most prior studies that demonstrated that rural-to-urban migrant workers had better physical health status than their non-migrating peers at home [35].

As our previous studies demonstrated, the healthy migrant effect existed in older rural-to-urban migrant workers in China [3], which referred to the lower mortality of older rural-to-urban migrant workers and was likely attributable to the self-selection for better health. Lu [36] argued that cities usually provide better health infrastructure and are more sustainable than the rural areas, which can provide older rural-to-urban migrant workers with better health services and may have a more positive impact on their health. Conversely, our findings found that migration for work in cities did not provide potential benefits of health service utilization for older rural-to-urban migrant workers in China, which is inconsistent with the assumption of New Economics of Labor Migration [7] and Lu [36]. A common finding in much of this literature is that, compared to older rural residents, older rural-to-urban migrant workers overall seemed to face substantial barriers to health service access. Some published studies [37] supported our views, Anh et al. found that migrant workers may benefit from better health service utilization in the local area, but the “healthy” effect can be reversed in the locality due to migration-related health risk factors. Furthermore, our findings corroborated the existence of barriers to health services for rural-to-urban migrant workers, as found in several previous studies using Chinese data [6,9]. Health services are provided by a medical institution on a fee-for-service basis, and there are massive differences in the cost of health services in rural and urban areas. Significantly, as the main component of China’s health system, the medical service system and public health system both undertake the complementary functions of treatment and prevention for old rural-to-urban migrant workers. As COVID-19 emphasizes, a truly systematic and effective public health system should focus on both institutional and technical measures, and special emphasis should be placed on the rural public health financing mechanism and improve the rural three-level public health service network. The sinking of health resources operates in favor of the accuracy and refinement of public health services in China, which is conducive to the long-term health of old rural-to-urban migrant workers.

As highlighted by several researchers [38,39], the health services in a community are becoming increasingly important, and our study suggested that attention should be paid to the relationship between the community and health service utilization, considering the heterogeneity of different dimensions. In terms of individual characteristics, significant influencing factors included educational attainment, income quantiles, self-assessed health, BMI, place of work, SAH, and the number of friends. In terms of health behavior, significant influencing factors included regular exercise every month. Our results may be useful to shed light on relevant health policy and public health interventions targeted to the vulnerable in China. Most studies [40] pointed out that health insurance influences the use of health services. Alternatively, it is worth noting that NCMS had no significant association with the utilization and the difference between the two groups in our study. Although the progress of off-site medical treatment in China is encouraging, much work remains to be undertaken regarding the health service utilization for older rural-to-urban migrant workers.

The results revealed the fact that 17.98% of the difference in two-week outpatient services utilization was caused by the observed influencing factors; however, the unobserved differences still accounted for a large proportion. Our results showed that there was no significant difference between two-week outpatient utilization. Moreover, 71.88% of the difference in inpatient services utilization was due to the observed influencing factors. The results demonstrated that income quantiles (49.57%), SAH (80.91%), and sex ratio in the community (−102.29%) were highly significant when explaining the differences in inpatient utilization. Among them, income quantiles have statistical significance to the socioeconomic difference. When combined with the high costs of physical examination and treatments for diseases due to ill-health, there can be catastrophic consequences for older rural-to-urban migrant workers, which may include falling into poverty or being pushed into deeper poverty. In general, the income problems faced by older rural-to-urban migrant workers cannot be blamed on their “not working hard” but is a social problem that governments should value, as public policy plays a very significant role in shaping income distribution and protecting their legitimate rights and interests, including protesting unpaid wages and raising the minimum wage. The positive and obvious function of such social policy is to give the poor people opportunities to change their life [41,42,43]. Echoing previously published studies [44,45], SAH contributed to the differences because SAH is largely a reflection of the integrated perception of their health, including the biological, psychological, and social dimensions that are strongly correlated with their health service utilization.

Although our study extended previous work in addressing some important challenges, it is not without limitations. First, CLDS 2016 is the latest data we can access, even if it is a little old. We will update our analysis in future studies as soon as the new data are released. In addition, CLDS 2016 is limited by its cross-sectional design that does not allow for the determination of time precedence or causal inferences between health service utilization and related factors. Second, we selected the sample from the CLDS 2016 data in accordance with our severe standards, so older rural-to-urban migrants were highly-selected sample groups, affecting the representativeness of the demographic distribution. Third, decomposition results are more obviously influenced by the explanatory variables. Many factors were not included in the decomposition model, such as medical costs and awareness of medical examination. Finally, the data used do not allow for the identification of returning rural-to-urban migrant workers who have migrated for at least 6 months but who have now returned; therefore, any hidden bias may remain.

## 5. Conclusions

Our study showed that older rural migrant workers had lower use of health services than older rural dwellers, and our results provided new empirical evidence on the influencing factors of health service utilization, and the differences among the two groups were diversified, especially contextual characteristics and individual characteristics. Our study may not only be referential for off-site medical settlement in China for older rural-to-urban migrant workers and also push for health policy reform in regards to the process of active aging in China.

## Figures and Tables

**Table 1:** Descriptive statistics of independent variables before and after CEM.

Variable	Before Matching *N* (%) Mean (SD)			After Matching *N* (%) Mean (SD)		
	Older Rural-to-Urban Migrant Workers	Older Rural Dwellers	*p*-Value *	Older Rural-to-Urban Migrant Workers	Older Rural Dwellers	*p*-Value #
Two weeks Outpatient	57 (5.99)	240 (8.93)	<0.01	48 (5.59)	118 (8.11)	<0.01
Inpatient	75 (7.88)	284 (10.61)	<0.05	65 (7.57)	131 (9.07)	<0.05
Individual characteristics						
Gender			<0.001			0.743
Men †	666 (69.96)	1448 (54.11)		617 (71.83)	822 (56.49)	
Women	286 (30.04)	1228 (45.89)		242 (28.17)	633 (43.51)	
Age			<0.001			0.892
50–54 †	506 (53.15)	848 (31.69)		469 (54.6)	551 (37.87)	
55–60	198 (20.8)	511 (19.1)		164 (19.09)	210 (14.43)	
61–65	248 (26.05)	1317 (49.22)		226 (26.31)	694 (47.7)	
Living arrangement			0.151			0.807
Live without spouse †	45 (4.73)	160 (5.98)		15 (1.75)	27 (1.86)	
Live with spouse	907 (95.27)	2516 (94.02)		844 (98.25)	1428 (98.14)	
Educational attainment			<0.05			0.771
Below primary school †	420 (44.12)	1750 (65.4)		391 (45.52)	971 (66.74)	
Primary school	365 (38.34)	731 (27.32)		333 (38.77)	395 (27.15)	
Middle school and above	167 (17.54)	195 (7.29)		135 (15.72)	89 (6.12)	
Political affiliation			<0.001			<0.001
Party members †	90 (9.45)	139 (5.19)		81 (9.43)	75 (5.15)	
The masses	862 (90.55)	2537 (94.81)		778 (90.57)	1380 (94.85)	
Type of industry						
Manufacturing and construction †	406 (44.76)	-		413 (48.08)	48.08	
Wholesale, retail trade, and catering	146 (16.1)	-		128 (14.9)	14.9	
Transportation and other non-agricultural sectors	355 (39.14)	-		318 (37.02)	37.02	
Farming	-	2676 (100)		-	1455 (100)	
Place of work						
Local †	458 (48.26)	-		650 (75.67)	75.67	
Out-of-town	491 (51.74)	-		209 (24.33)	24.33	
Working hours						
Moderate labor †	724 (76.05)	-		408 (47.5)	47.5	
Excessive labor	228 (23.95)	-		451 (52.5)	52.5	
NCMS			<0.01			<0.01
Yes †	869 (91.28)	2501 (93.46)		781 (90.92)	1372 (94.3)	
None	83 (8.72)	175 (6.54)		78 (9.08)	83 (5.7)	
Basic endowment scheme			< 0.05			<0.01
Yes †	878 (92.23)	2540 (94.92)		794 (92.43)	1390 (95.53)	
None	74 (7.77)	136 (5.08)		65 (7.57)	65 (4.47)	
Income quantiles			0.326			0.897
Poorest †	20	2.1		48 (22.33)	226 (20.79)	
Poorer	40	4.2		42 (19.53)	214 (19.69)	
Middle	154	16.18		50 (23.26)	224 (20.61)	
Richer	333	34.98		30 (13.95)	236 (21.71)	
Richest	405	42.54		45 (20.93)	187 (17.20)	
SAH			<0.001			0.833
Good †	583 (61.24)	1159 (43.31)		538 (62.63)	709 (48.73)	
Fair	276 (28.99)	821 (30.68)		246 (28.64)	461 (31.68)	
Poor	93 (9.77)	696 (26.01)		75 (8.73)	285 (19.59)	
BMI			<0.001			0.916
Underweight †	42 (4.41)	282 (10.54)		22 (2.56)	57 (3.92)	
Ideal	535 (56.2)	1575 (58.86)		501 (58.32)	948 (65.15)	
Overweight	375 (39.39)	819 (30.61)		336 (39.12)	450 (30.93)	
Number of friends			0.078			0.398
≤5 †	543 (57.04)	1628 (60.84)		491 (57.16)	858 (58.97)	
6~10	226 (23.74)	551 (20.59)		201 (23.4)	312 (21.44)	
≥11	183 (19.22)	497 (18.57)		167 (19.44)	285 (19.59)	
Health behavior						
Smoking			<0.001			<0.05
Yes †	331 (34.77)	631 (23.58)		396 (46.1)	349 (23.99)	
No	621 (65.23)	2045 (76.42)		463 (53.9)	1106 (76.01)	
Drinking			<0.001			0.099
Yes †	320 (33.61)	697 (26.05)		288 (33.53)	394 (27.08)	
No	632 (66.39)	1979 (73.95)		571 (66.47)	1061 (72.92)	
Regular exercise every month			<0.001			<0.05
Yes †	232 (24.37)	510 (19.06)		202 (23.52)	292 (20.07)	
No	720 (75.63)	2166 (80.94)		657 (76.48)	1163 (79.93)	
Health outcome						
Sense of fairness			0.246			0.152
Unhappy †	58 (6.09)	204 (7.62)		49 (5.7)	87 (5.98)	
Fair	283 (29.73)	809 (30.23)		248 (28.87)	430 (29.55)	
Happy	611 (64.18)	1663 (62.14)		562 (65.42)	938 (64.47)	
Contextual characteristic						
Proportion of ethnic minorities (%)	3.99 (15.35)	10.61 (25.37)	<0.01	4.17 (15.95)	8.34 (19.15)	<0.05
Sex ratio in the community (%)	1.04 (1.78)	1.62 (6.08)	<0.001	1.01 (1.54)	1.91 (8.03)	<0.05
Number of health facilities per capita in the community	0.01 (0.01)	0.01 (0.01)	<0.001	0.01 (0.01)	0.01 (0.02)	<0.05
Service quality index of the community	0.66 (1.31)	0.49 (1.31)	<0.001	−0.01 (0.09)	−0.06 (0.01)	<0.01
Service quality index of the city	0.80 (1.67)	0.57 (1.12)	<0.001	0.09 (0.81)	0.08 (0.03)	<0.01
Health index of the community population	0.69 (0.64)	1.02 (0.61)	<0.01	0.01 (0.21)	0.01 (0.31)	<0.01
Region			<0.001			<0.05
East †	55 (21.24)	1071 (40.02)		612 (71.25)	594 (40.82)	
Middle	60 (23.17)	851 (31.8)		147 (17.11)	488 (33.54)	
West	144 (55.6)	754 (28.18)		100 (11.64)	373 (25.64)	
City level			<0.01			0.08
Sub-provincial city and above †	71 (16.82)	497 (18.57)		123 (14.32)	307 (21.1)	
Below sub-provincial city	108 (25.59)	2179 (81.43)		736 (85.68)	1148 (78.9)	
L_1_	0.6372			<0.0001		

Note: † Reference levels in the regressions; virtual variables for chi-square test; * *p*-value indicated the actual *p*-values after matching; # *p*-value indicated the weight to be considered; *N* (%) were reported. NCMS: New cooperative medical scheme. SAH: self-assessed of health status.

**Table 2:** Association of independent variables and health service utilization in logit regression analysis.

Variable	Two Weeks Outpatient				Inpatient			
	Older Rural-to-Urban Migrant Workers		Older Rural Dwellers		Older Rural-to-Urban Migrant Workers		Older Rural Dwellers	
Individual characteristics								
Gender								
Men †								
Women	−0.2605	0.4764	0.1698	0.2641	0.4459	0.4378	−0.1142	0.2441
Age								
50–54 †								
55–60	−1.3506 *	0.679	−0.4980	0.379	0.2396	0.4238	0.0254	0.3387
61–65	−0.3351	0.4718	−0.2933	0.2662	0.4382	0.3961	0.2193	0.2463
Living arrangement								
Live with spouse †								
Live without spouse	0.3737	1.3539	0.1723	0.7797	−0.8918	0.82	0.4313	0.773
Educational attainment								
Below primary school †								
Primary school	0.3199	0.4144	0.1719	0.2829	0.3042	0.3642	−0.5742 *	0.2851
Middle school and above	0.2252	0.6897	−0.2361	0.7659	−0.0760	0.529	−1.3347 *	0.7577
Political affiliation								
Party members †								
The masses	0.3737	1.3539	1.4445	1.0397	0.3147	0.5688	0.2126	0.5518
Type of industry								
Manufacturing and construction †			-	-			-	-
Wholesale, retail trade and catering	0.2284	0.4881	-	-	0.0088	0.4325	-	-
Transportation and other non-agricultural sectors	−0.4293	0.4437	-	-	0.0903	0.3556	-	-
Farming	-		-	-	-		-	-
Place of work								
Local †			-	-			-	-
Out-of-town	−0.4509	0.4723	-	-	−0.9231 **	0.4394	-	-
Working hours								
Moderate labor †			-	-			-	-
Excessive labor	0.058	0.3842	-	-	0.5263	0.3224	-	-
Medical scheme								
Yes †								
None	0.9745	0.9632	−0.4634	0.743	0.0567	0.8505	−0.3626	0.6252
NCMS								
Yes †								
None	−0.2336	1.0771	−0.8543	0.9036	−0.6618	0.976	0.034	0.6824
Income quantiles								
Poorest †								
Poorer	−0.1935	0.5214	−0.2197 *	0.3057	0.0848	0.4711	−0.1497	0.2972
Middle	−0.5663	0.5753	−0.0829 *	0.3082	−0.0343	0.5107	−0.1875	0.3074
Richer	0.1037	0.5903	−0.4252	0.3538	0.723	0.5117	0.1213	0.3148
Richest	−1.3272	0.7641	−0.5246	0.4252	1.0054	0.5033	0.1817	0.3524
SAH								
Good †								
Fair	0.5303	0.4561	0.9238 ***	0.3037	0.7800 **	0.3511	0.8786 ***	0.2685
Poor	2.5872 ***	0.477	2.0299 ***	0.2974	2.3718 ***	0.4031	1.7987 ***	0.2707
BMI								
Underweight †								
Ideal	0.3076	0.3769	−0.7318 **	0.4083	−0.8318	0.6449	0.5406	0.5051
Overweight	0.3994	1.2811	−0.7271	0.4433	−1.5398	0.6774	0.2122	0.5372
Number of friends								
≤5 †								
6~10	−0.5572	0.5049	0.33	0.2504	0.1084	0.3899	0.7072 **	0.2305
≥11	0.2666	0.5337	−0.2957	0.3089	0.4095	0.3968	0.2182	0.2659
Health behavior								
Smoking				<0.001				<0.05
Yes †								
No	0.1269	0.4569	0.5024	0.3139	−0.0830	0.3715	0.2018	0.275
Drinking								
Yes †								
No	0.9937	0.5225	0.2418	0.2987	−0.4316	0.367	0.1285	0.2569
Regular exercise every month								
Yes †								
No	−0.6749	0.3976	−0.0720	0.2891	−0.8280 **	0.326	0.0099	0.2668
Health outcome								
Sense of fairness								
Unhappy †								
Fair	0.2501	0.6492	−0.9945 *	0.3519	−1.2225 **	0.5714	−0.6119	0.3633
Happy	−0.8565	0.6635	−0.7262 **	0.3299	−0.7139	0.5209	−0.3945	0.3457
Contextual characteristic								
Proportion of ethnic minorities	−0.0113	0.0134	0.0016	0.0041	0.01	0.0089	0.0032	0.0037
Sex ratio in the community	−1.0148	0.7023	−0.0088	0.0269	−0.0876	0.2968	0.0192	0.0099
Number of health facilities per capita in the community	−158.2989	235.357	11.246	74.8736	−20.7497	178.8096	16.2103	66.6137
Service quality index of the community	0.3742	0.3038	−0.3039	0.2366	0.1196	0.2612	−0.0969 *	0.2069
Service quality index of the city	0.3298	0.2225	−0.0079	0.1764	0.1004	0.1946	−0.0895	0.1746
Health index of the community population	−0.3254	0.4373	−0.2828	0.1864	0.0713	0.2406	−0.1125	0.1328
Region								
East †								
Middle	0.3376	0.557	−0.2886	0.2875	0.4398	0.4448	−0.4139	0.2719
West	0.6087	0.5788	0.0615	0.2638	0.2092	0.5073	0.1955	0.2379
City level								
Sub-provincial city and above †								
Below sub-provincial city	2.2806 *	1.165	0.0275	0.2803	0.0476	0.4482	0.0572	0.2565

Note: † Reference levels in the regressions. The Symbol of “*” is defined by a *p* value < 0.05; the Symbol of “**” is defined by a *p* value < 0.01; the Symbol of “***” is defined by a *p* value < 0.001. NCMS: New cooperative medical scheme. SAH: self-assessed of health status.

**Table 3:** Fairlie’s decomposition of the difference of health service utilization between matched older rural-to-urban migrant workers and older rural dwellers.

Terms of Decomposition	Two Weeks Outpatient			Inpatient		
Total gap (%)	−0.0082			−0.0106		
Explained (%)	17.98%			71.88%		
Explained						
Variable	Contribution (%)	95%CI		Contribution (%)	95%CI	
Individual characteristics						
Age	−4.44	−0.0028	0.0035	−0.30	−0.0014	0.0015
Gender	5.27	−0.0032	0.0023	27.91	−0.0081	0.0021
Living arrangement	−0.95	−0.0009	0.0010	0.57	−0.0007	0.0006
Educational level	0.16	−0.0014	0.0014	39.20	−0.0084	0.0000
Income quantiles	−25.26	−0.0019	0.0060	49.57 *	−0.0103	−0.0003
NCMS	14.61	−0.0071	0.0047	8.29	−0.0076	0.0059
Basic endowment scheme	−5.31	−0.0035	0.0044	2.84	−0.0045	0.0039
Political affiliation	−9.86	−0.0006	0.0023	7.14	−0.0029	0.0014
Number of friends	7.77	−0.0025	0.0012	7.80	−0.0028	0.0012
SAH	0.92	−0.0045	0.0043	80.91 ***	−0.0139	−0.0033
BMI	−5.35	−0.0015	0.0023	−16.12	−0.0007	0.0042
Health behavior						
Smoking	1.76	−0.0024	0.0021	−17.59	−0.0016	0.0054
Drinking	−2.41	−0.0016	0.0020	0.27	−0.0013	0.0012
Regular exercise every month	7.47	−0.0030	0.0018	−3.15	−0.0013	0.0020
Health outcome						
Sense of fairness	−0.63	−0.0017	0.0018	6.31	−0.0038	0.0024
Contextual characteristic						
Proportion of ethnic minorities	−0.49	−0.0016	0.0017	2.36	−0.0014	0.0009
Sex ratio in the community	2.99	−0.0016	0.0011	−102.29 ***	0.0065	0.0152
Number of health facilities per capita in the community	3.62	−0.0028	0.0022	4.45	−0.0027	0.0018
Service quality index of the community	30.10	−0.0093	0.0043	−6.05	−0.0043	0.0056
Service quality index of the city	18.20	−0.0074	0.0044	−1.80	−0.0074	0.0078
Health index of the community population	30.56	−0.0070	0.0020	−7.78	−0.0034	0.0051
Region	−50.68	−0.0045	0.0128	−9.46	−0.0083	0.0103
City level	−4.88	−0.0018	0.0026	2.93	−0.0016	0.0010

Note: Total gap referred to the utilization rate of health service of older rural-to-urban migrant workers minus that of older rural dwellers. The Symbol of “*” is defined by a *p* value < 0.05; the Symbol of “***” is defined by a *p* value < 0.001. NCMS: New cooperative medical scheme. SAH: self-assessed of health status.

## Data Availability

The datasets used in this study are available from the 2016 CLDS of Center for Social Survey, Sun Yat-sen University (available online: http://css.sysu.edu.cn/Data; accessed on 15 May 2022).

## Abbreviations

CLDS	China Labor-Force Dynamic Survey
CEM	coarsened exact matching method
Hukou	Chinese household registration system
NCMS	New cooperative medical scheme
SAH	self-assessed of health status
95%CI	95% Coefficient Interval

## References

[1] The National Bureau of Statistics (2020). Survey Report on Rural-to-Urban Migrants. http://www.stats.gov.cn/tjsj/zxfb/202004/t20200430_1742724.html.

[2] Wang T.T., Dds M., Neeraj M.A. (2019). The Graying of America: Considerations and Training Needs for Geriatric Patient Care-ScienceDirect. J. Oral Maxillofac. Surg..

[3] Li D., Zhou Z., Shen C., Zhang J., Yang W., Nawaz R. (2020). Health Disparity between the Older Rural-to-Urban Migrant Workers and Their Rural Counterparts in China. Int. J. Environ. Res. Public Health.

[4] Li D., Zhu L., Zhang J., Yang J. (2021). Decomposing Differences of Health Service Utilization among Chinese Rural Migrant Workers with New Cooperative Medical Scheme: A Comparative Study. Int. J. Environ. Res. Public Health.

[5] Wu M., Duan C., Zhu X. (2016). Effect of Social Support on Psychological Well-being in Elder Rural-urban Migrants. Popul. J..

[6] Zhao X., Min D., Ma W. (2015). Utilization of outpatient service and influential factors of expenditure of middle-aged and elderly migrant workers in China. J. Peking Univ..

[7] Stark O., Taylor J.E. (1991). Migration Incentives, Migration Types: The Role of Relative Deprivation. Econ. J..

[8] Jiménez-Rubio D., Hernández-Quevedo C. (2011). Inequalities in the use of health services between immigrants and the native population in Spain: What is driving the differences?. Eur. J. Health Econ..

[9] Ahad F.B., Zick C.D., Simonsen S.E., Mukundente V., Davis F.A., Digre K. (2019). Assessing the Likelihood of Having a Regular Health Care Provider among African American and African Immigrant Women. Ethn. Dis..

[10] Xin Y. (2018). Difference in utilization of basic public health service between registered and migrant population and its related factors in China. Chin. J. Public Health.

[11] Zheng L., Hu R., Dong Z., Hao Y. (2018). Comparing the needs and utilization of health services between urban residents and rural-to-urban migrants in China from 2012 to 2016. BMC Health Serv. Res..

[12] Puszczalowska-Lizis E., Koziol K., Omorczyk J. (2021). Perception of footwear comfort and its relationship with the foot structure among youngest-old women and men. PeerJ.

[13] Mark G., Subramanian S.V., Daniel V., Danny D. (2015). Internal migration, area effects and health: Does where you move to impact upon your health?. Soc. Sci. Med..

[14] Andersen R., Rice T.H., Kominski G. (2007). Changing the U. S. Health Care System: Key Issues in Health Services, Policy, and Management. JAMA J. Am. Med. Assoc..

[15] Aday L., Awe W., Gochman D. (1997). Health Services Utilization Model. Handbook of Health Behavior Research.

[16] Hajek A., Bock J.O., König H.H. (2017). Which factors affect health care use among older Germans? Results of the German ageing survey. BMC Health Serv. Res..

[17] Lu S., Le Y. (2018). Anderson Health Service Utilization Behavior Model: Interpretation and Operationalization of Indicator System. Chin. Health Econ..

[18] Chen M. (2018). Andson’s behavioral model of Health Service Utilization and its application. J. Nanjing Med. Univ..

[19] Yu J., Xu H., Wang L. (2020). Local Government Finance expends, official appointment and investment synchronism. Manag. Rev..

[20] Li D., Zhou Z., Si Y., Xu Y., Shen C., Wang Y., Wang X. (2018). Unequal distribution of health human resource in mainland China: What are the determinants from a comprehensive perspective?. Int. J. Equity Health.

[21] China’s National Bureau of Statistics Provisions on the Statistical Division of Urban and Rural Areas. http://www.stats.gov.cn/tjsj/pcsj/rkpc/5rp/html/append7.htm.

[22] Yip W., Hsiao W.C. (2008). The Chinese Health System at A Crossroads. Health Aff..

[23] Centre for Health Statistics and Information (2004). Research on National Health Services: An Analysis Report of National Health Services Survey in 2003.

[24] Ministry of Health of the People’s Republic of China (2009). An Analysis Report of National Health Services Survey in China.

[25] Gelberg L., Andersen R.M., Leake B.D. (2000). The Behavioral Model for Vulnerable Populations: Application to Medical Care Use and Outcomes for Homeless People. Health Serv. Res..

[26] Sidney J.A., Coberley C., Pope J.E., Wells A. (2015). Extending coarsened exact matching to multiple cohorts: An application to longitudinal well-being program evaluation within an employer population. Health Serv. Outcomes Res. Method.

[27] Ho D.E., Imai K., Stuart K.E.A. (2007). Matching as nonparametric preprocessing for reducing model dependence in parametric causal inference. Political Anal..

[28] Iacus S.M., King G., Porro G. (2012). Causal inference without balance checking: Coarsened exact matching. Political Anal..

[29] Iacus S.M., King G., Porro G. (2011). Multivariate Matching Methods That Are Monotonic Imbalance Bounding. J. Am. Stat. Assoc..

[30] Gotsadze G., Murphy A., Shengelia N., Zoidze A. (2015). Healthcare utilization and expenditures for chronic and acute conditions in Georgia: Does benefit package design matter?. BMC Health Serv. Res..

[31] Hametner C., Kellert L., Ringleb P.A. (2015). Impact of sex in stroke thrombolysis: A coarsened exact matching study. BMC Neurol..

[32] Blackwell M., Iacus S., King G. (2009). cem: Coarsened exact matching in Stata. Stata J..

[33] Fagbamigbe A.F., Morakinyo O.M., Balogun F.M. (2022). Sex inequality in under-five deaths and associated factors in low and middle-income countries: A Fairlie decomposition analysis. BMC Public Health.

[34] Alieu S., Klara J. (2020). Disentangling the rural-urban immunization coverage disparity in The Gambia: A Fairlie decomposition. Vaccine.

[35] Chen J. (2011). Internal migration and health: Re-examining the healthy migrant phenomenon in china. Soc. Sci. Med..

[36] Lu Y. (2010). Rural-urban migration and health: Evidence from longitudinal data in Indonesia. Soc. Sci. Med..

[37] Le Kim A.T., Pham L.T., Vu L.H., Schelling E. (2012). Health services for reproductive tract infections among female migrant workers in industrial zones in Ha Noi, Viet Nam: An in-depth assessment. Reprod. Health.

[38] Mohammadi Bidhandi H., Patrick J., Noghani P., Varshoei P. (2019). Capacity planning for a network of community health services. Eur. J. Oper. Res..

[39] Hill J.D., Cuthel A.M., Grudzen C.R. (2020). Access to Home and Community Health Services for Older Adults With Serious, Life-Limiting Illness: A Study Protocol. Am. J. Hosp. Palliat. Med..

[40] Wang J., Pei Y., Zhong R., Wu B. (2020). Outpatient Visits among Older Adults Living Alone in China: Does Health Insurance and City of Residence Matter?. Int. J. Environ. Res. Public Health.

[41] Li D., Zhou Z., Zhao D. (2022). Health inequality among rural elder residents in Shaanxi province. Chin. J. Public Health.

[42] Crettaz E. (2010). Alleviating Working Poverty in Postindustrial Economies.

[43] Liu J. (2017). Working Poor in Mainland China: Concept and Life Trajectory of Its Main Working Groups. J. US-China Public Adm..

[44] Borrell C., Espelt A., Rodriguez-Sanz M., Burström B., Muntaner C., Pasarín M.I., Benach J., Marinacci C., Roskam A.J., Schaap M. (2009). Analyzing differences in the magnitude of socioeconomic inequalities in self-perceived health by countries of different political tradition in Europe. Int. J. Health Serv..

[45] Lu L., Zeng J., Zeng Z. (2017). What limits the utilization of health services among china labor force? analysis of inequalities in demographic, socio-economic and health status. Int. J. Equity Health.

